# Patterns of alcohol drinking and its association with obesity: data from the third national health and nutrition examination survey, 1988–1994

**DOI:** 10.1186/1471-2458-5-126

**Published:** 2005-12-05

**Authors:** Ahmed A Arif, James E Rohrer

**Affiliations:** 1 Texas Tech University Health Sciences Center, Department of Family & Community Medicine, Division of Health Services Research, Lubbock, TX, USA; 2 Mayo Clinic Family Medicine Program/Rochester, Department of Family Medicine, 406 West Main Street, Kasson MN, USA

## Abstract

**Background:**

Recent reports suggest that alcohol use may have a protective effect on obesity. This study explores association between obesity and alcohol consumption in the non-smoking U.S. adult population.

**Methods:**

We analyzed data on a total of 8,236 respondents who participated in the Third National Health and Nutrition Examination Survey. Body mass index (weight-kg/height-m^2^) was derived from measured height and weight data and categorized into: normal weight, overweight, and obese. Alcohol consumption was measured using following measures: history of drinking, binge drinking, quantity of drinks/day, frequency of drinking, and average volume of drinks/week.

**Results:**

Mean body mass index in this sample of non-smokers was 26.4 (95% CI: 26.1, 26.7). Approximately 46% of respondents were classified as current drinkers. Current drinkers had lower odds of obesity (Adjusted odds ratio = 0.73, 95% CI: 0.55, 0.97) as compared to non-drinkers. The odds of overweight and obesity were significantly greater among binge drinkers and those consuming four or more drinks/day. However, those who reported drinking one or two drinks per day had 0.46 (95% CI: 0.34, 0.62) and 0.59 (95% CI: 0.41, 0.86) times the odds of obesity, respectively. Similarly, the odds of obesity were significantly lower among those who reported drinking frequently and consuming less than five drinks per week. The association between overweight and other alcohol measures was less pronounced.

**Conclusion:**

The results suggest further exploring the possible role of moderate alcohol drinking in controlling body weight in adults.

## Background

The proportion of the population that is overweight or obese has increased steadily in many developed nations including the U.S. [1,2]. The continual rise in the prevalence of obesity is of concern to public health agencies because of its potentially explosive effect on the health of individuals and the cost to the health care system [3,4]. Alcohol consumption is common in the U.S., with the past-month prevalence of consuming at least one drink is reported at fifty percent among 12 years and older [5]. Average intake of alcohol among adults in the U.S. is approximately 10% of the total daily energy intake [6]. Alcohol, which is the second most energy dense macronutrient consumed [7], is known to reduce oxidation of fat and favors fat storage which may result in weight gain [8]. However, previous studies have shown that despite the added calories, alcohol drinkers do not gain extra weight as compared to non-drinkers [6]. Other studies have reported a J-shaped relationship between alcohol consumption and body mass index and waist-to-hip ratio such that light to moderate drinking has beneficial effect in reducing weight whereas non-drinking and heavy or risky drinking having the opposite effect [9,10]. However, the inverse association between alcohol drinking and weight gain has been prominent among women with the results among men remaining inconclusive [9,11-14]. The aims of this study were to assess the cross-sectional relationship between alcohol drinking and obesity in a nationally representative sample of non-smoking adults, using different measures of alcohol consumption.

## Methods

### Sample selection

The National Health and Nutrition Examination Survey III is a large cross-sectional survey of non-institutionalized U.S. population conducted between 1988 and 1994 [15]. Data for this study were extracted from two data files: the adult questionnaire and the physical examination data file. Initially, a total of 19,618 respondents 18 years or older were selected. Those who were pregnant (n = 322) or whose information on lifetime drinking (n = 2850) or drinking in the previous 12 months (n = 2) were missing were excluded from the analysis. The analysis was limited only to lifetime non-smokers leaving a final sample size of 8,236.

### Variables

The main dependent variable was body mass index (BMI = Weight in kg/height in m^2^). Weight and height were measured during physical examination of study participants. BMI was recorded from the physical examination data file and categorized into normal weight (BMI<25 kg/m^2^), overweight (BMI 25 kg/m^2^–29.9 kg/m^2^) and obese (BMI ≥30 kg/m^2^).

The main independent variable of interest was alcohol consumption. Alcohol consumption was measured using these questions: 1) History of drinking: *in your entire life, have you had at least 12 drinks of any kind of alcoholic beverage *(variable MAPE1)? *In the past 12 months did you have at least 12 drinks of any kind of alcoholic beverage *(variable MAPE2)?; 2) Quantity of drinking: *on the average, on the days that you drank alcohol, how many drinks did you have a day *(variable MAPE4)?, those consuming four or more drinks were classified as heavy drinkers; 3) Frequency of drinking (Number of drinking days): *in the past 12 months, how many days of the year did you drink any alcoholic beverages *(variable MAPE3s)?; 4) Average volume of drinking alcoholic beverages = frequency*quantity/52, and 5) Binge drinking: *In the past 12 months, how many days of the year did you have 5 or more drinks on a single day *(variable MAPE6S)? The variables MAPE1 and MAPE2 were used to classify respondents to non-drinker, ex-drinker, and current drinker categories. A drink was considered as a 12-oz beer, a 4-oz glass of wine, or an ounce of liquor. In NHANES III no distinction was made between different types of alcoholic drinks. The covariates included in the model were gender, age, race/ethnicity, marital status, poverty income ratio, education, rural/urban, self-rated health, and leisure time physical activity.

### Statistical analysis

Descriptive statistics were used to describe the characteristics of the study sample by drinking status. The association between weight status and risky alcohol drinking, which included both heavy drinking and binge drinking, was modeled using logistic regression analysis. The association between BMI and different measures of alcohol consumption was assessed using chi-square statistics. Multinomial logistic regression analyses were used to assess relationship between the categorical BMI variable and alcohol consumption adjusted for all the covariates. In a multinomial logistic regression model the odds of associations for both the overweight and obese categories were compared simultaneously to the normal weight category which was used as a common referent. Odds ratios and 95 percent confidence intervals were computed and adjusted for the confounding effects of age, sex, race/ethnicity, poverty income ratio, education, marital status, rural/urban, self-rated health, and leisure time physical activities. Because of the complex survey design, STATA statistical software version 8.2 (College Station, TX), which allows incorporation of sampling weights, strata, and primary sampling units, was used to perform all the analyses.

## Results

The overall prevalence of overweight (25 kg/m^2^<BMI< 30 kg/m^2^) and obesity (BMI≥30 kg/m^2^) in this sample of never smokers was 31.4 percent (95% CI: 29.5, 33.4) and 21.9 percent (95% CI: 20.0, 23.8), respectively. Although men had the highest prevalence of overweight 39.6 percent (95% CI: 36.1, 43.1), the prevalence of obesity was highest among women 24.9 percent (95% CI: 22.3, 27.4). Non-Hispanic Blacks had the highest prevalence of obesity (33.0 percent, 95% CI: 30.5, 35.5).

Approximately 46 percent of the sample was categorized as current drinkers; about ten percent were classified as heavy drinkers, consuming four or more drinks per day (Table 1). The prevalence of current drinking was almost twice among male non-smokers as compared to female non-smokers (63 percent vs 35 percent, *p *< 0.001). The mean (geometric mean) number of alcohol drinks consumed per day by the respondents was 2.3; 2.8 drinks/day among men and 1.9 drinks/day among women. Almost one-third of obese individuals were current drinkers.

**Table 1:** Characteristics of the study sample by drinking status- the third National Health and Nutrition Examination Survey, 1988–1994

**Variables**	**Non-Drinkers**	**Ex-Drinkers**	**Current Drinkers**
	**(n = 2539) %**^**a**^	**(n = 2755) %**^**a**^	**(n = 2942) %**^**a**^

**All**	**24.1**	**30.0**	**45.9**
**Gender**			
Female	30.9	33.8	35.4
Male	13.6	23.9	62.5
**Age**			
18–29	24.2	21.3	54.5
30–39	14.0	27.5	58.6
40–49	22.2	32.1	45.7
50–59	26.1	34.2	39.7
60–69	27.9	38.0	34.1
70+	41.3	42.2	16.5
**Race/Ethnicity**			
Non-Hispanic White	19.9	29.7	50.4
Non-Hispanic Black	31.7	35.0	33.3
Hispanic	28.8	29.6	41.6
Other Race/Ethnicity	59.2	19.6	21.2
**Poverty Income Ratio**			
Above Poverty	22.5	29.8	47.7
Below Poverty	37.5	30.7	31.8
**Education**			
High School or more	19.5	29.2	51.3
Less than High School	41.0	32.6	26.5
**Marital Status**			
Married/Living as Married	22.5	32.0	45.5
Widowed/Divorced/Separated	28.0	38.2	33.8
Never Married	25.4	19.4	55.2
**Location**			
Rural	29.1	32.3	38.6
Urban	19.6	27.7	52.7
**Health**			
Excellent/Very Good/ Good	22.1	28.3	49.6
Fair/Poor	37.3	39.7	23.0
**Leisure Time Physical Activity**^**b**^			
First Quartile (none)	40.2	34.2	25.6
Second Quartile (1–9)	24.7	32.5	42.8
Third Quartile (10–30)	19.4	30.2	50.4
Fourth Quartile (>30)	19.2	24.2	56.5
**Body Mass Index**^**c**^			
Normal Weight	23.5	25.8	50.7
Overweight	22.1	31.3	46.6
Obese	28.4	36.6	35.0

^a ^Weighted percent

^b ^Nine leisure time physical activities (walking, jogging/running, bicycle, swimming, aerobics, other dancing, calisthenics/exercises, garden/yard work, weight lift) were summed and categorized into four quartiles.

^c ^Normal Weight (BMI <25 kg-m^2^), Overweight (25 kg-m^2^<BMI< 30 kg-m^2^), Obese (BMI ≥ 30 kg-m^2^)

Table 2 presents the cross-sectional relationship between obesity and drinking patterns. The odds of obesity among current drinkers were 0.73 times lower than the odds among non-drinkers (95%CI: 0.55, 0.97). Significantly greater odds of overweight and obesity were observed among those engaged in binge drinking. Similarly, those who reported drinking four or more drinks per day had 30 percent greater odds of being overweight (95%CI: 1.00, 1.68) and 46 percent greater odds of obesity (95%CI: 0.98, 2.17); however respondents who reported drinking one or two drinks/day had significantly lower odds of obesity. When examining the frequency of drinking days, significantly lower odds of obesity were observed among respondents in higher quartiles. Similarly, those who consumed less than five drinks/week had 0.62 times reduce odds of obesity (95%CI: 0.46, 0.82) as compared to non/ex-drinkers (Table 2). The results were less pronounced in the overweight category.

**Table 2:** Multiple logistic regression analysis of overweight and obesity among never smokers- the third National Health and Nutrition Examination Survey, 1988–1994

	**Overweight**		**Obese**	
	**Odds Ratio**^**a**^	**95% CI**	**Odds Ratio**^**a**^	**95% CI**

**History of Drinking**				
Non-Drinker	1.00		1.00	
Ex-Drinker	1.14	(0.90–1.45)	1.10	(0.88–1.37)
Current Drinker	0.98	(0.71–1.35)	0.73	(0.55–0.97)
				
**Binge Drinking**				
No Binge drinking	1.00		1.00	
Yes Binge drinking	1.45	(1.02–2.05)	1.77	(1.18–2.65)
Non/Ex-Drinker	1.27	(0.95–1.71)	1.80	(1.30–2.50)
				
**Quantity of Drinks/day**				
Non/Ex-Drinker	1.00		1.00	
1 drink/day	0.71	(0.53–0.95)	0.46	(0.34–0.62)
2 drinks/day	0.83	(0.61–1.14)	0.59	(0.41–0.86)
3 drinks/day	1.40	(0.87–2.26)	1.07	(0.64–1.79)
4 or more drinks/day	1.30	(1.00–1.68)	1.46	(0.98–2.17)
				
**Number of drinking days/year**				
Non/Ex-Drinker	1.00		1.00	
1^st ^quartile	1.06	(0.75–1.50)	1.02	(0.69–1.52)
2^nd ^quartile	0.75	(0.59–0.96)	0.49	(0.35–0.68)
3^rd ^quartile	0.99	(0.75–1.29)	0.64	(0.47–0.88)
4^th ^quartile	0.84	(0.60–1.18)	0.61	(0.38–0.99)
				
**Average Volume of Drinks/week**^**b**^				
Non/Ex-Drinker	1.00		1.00	
< 5 drinks/week	0.84	(0.65–1.08)	0.62	(0.46–0.82)
5–9 drinks/week	1.08	(0.73–1.58)	0.79	(0.51–1.23)
10–14 drinks/week	1.03	(0.60–1.76)	1.24	(0.66–2.36)
15 or more drinks/week	1.49	(0.98–2.26)	1.10	(0.63–1.91)

^a ^Adjusted for age, sex, race/ethnicity, poverty income ratio, education, marital status, rural/urban, self-rated health, and leisure time physical activities. Nine leisure time physical activities (walking, jogging/running, bicycle, swimming, aerobics, other dancing, calisthenics/exercises, garden/yard work, weight lift) were summed and categorized into four quartiles.

^b ^Average volume of drinking alcoholic beverages = frequency*quantity/52.

## Discussion

This cross-sectional study found an inverse relationship between moderate consumption of alcohol and obesity in a large representative sample of non-smoking U.S. adults. Current drinkers had the lowest odds of obesity. A dose-response relationship was observed with increasing quantity of drinks and odds of obesity. Study participants reporting drinking one or two drinks per day had lower odds of obesity. The association was less pronounced in the overweight category.

The major limitation of our study lies in its cross-sectional nature which precludes establishing any cause and effect relationship. The strength of our study was that it includes anthropometric measurements of body mass index which makes our results more reliable and avoids potential bias that may result from self-reported height and weight data. However, we note that our findings confirm those reported in studies using self-reported height and weight. We used different measures of alcohol drinking habits to illuminate the role of alcohol drinking on obesity, which only few of the cross-sectional studies have done before. However, the accuracy of self-reported data on alcohol consumption is subject to debate. Assuming under-reporting of alcohol consumption by study respondents, it is likely that our study results are biased downwards. Measurement of alcohol use also has not been consistent in epidemiological studies. Differences in measurements can make cross-study comparisons difficult. Our study was limited to persons who never smoked; hence findings are not generalizable to smokers or past smokers. Use of odds ratio, instead of prevalence ratio, as an effect measure in a cross-sectional study of diseases of high prevalence has been shown to overestimate the true association [16]. Statistical methods to estimate prevalence ratio and its variance in a survey sample using a categorical outcome variable are not well developed; hence the odds ratio remain the effect measure of choice in such studies.

In this study, the odds of overweight and obesity were significantly higher among those who indulged in binge drinking and/or heavy drinking (consuming four or more drinks/day). In contrast, light-to-moderate drinking (consuming one or two drinks/day) was associated with lower odds of overweight and obesity. We observed a J-shape pattern, reported by others [9,10] among women binge drinkers and both men and women heavy drinkers (consuming four or more drinks/day). The odds of obesity were twice (Adjusted OR = 2.36, 95%CI: 1.58, 3.53) as likely among women binge drinkers and non/ex-drinkers (Adjusted OR = 2.09, 95%CI: 1.46, 2.99) as compared to non-bingers. No significant association was found among overweight women or overweight and/or obese men. For heavy drinkers, the adjusted odds of obesity were 2.40 (95%CI: 1.23, 4.58) for women and 2.25 (95%CI: 1.45, 3.48) for men heavy drinkers and 1.82 (95%CI: 1.32, 2.51) for women and 1.52 (95%CI: 1.00, 2.30) for men non/ex-drinkers, as compared to those who consumed less than fours drinks/day. In our earlier study of primary care patients we did not observe any association between obesity and binge drinking, possibly due to a small sample size [13]. Binge drinking is considered as high risk drinking and consumption of a high amount of alcohol has been associated with increased morbidity, mortality, and poor self-rated health [17-19]; approximately 8% of the U.S. adult population engages in risky drinking behavior [20]. In a prospective study of British adults, Wannamethee and Shaper [18], reported that heavy alcohol drinkers (≥ 30 g/d) had the highest prevalence of weight gain and obesity, irrespective of the type of alcohol consumed.

Other studies have shown an inverse association between increasing quantity of alcohol consumption and weight gain. In a study of Danish adults, Vadstrup et al [21] found an inverse association of waist circumference (measured ten years after the baseline) with total drinks of wine consumed per week. Those who consumed one to seven drinks per week had smallest waist circumference. No information on other measures of drinking was available. In a randomized controlled trial of German adults, Flechtner-Mors et al. [22] evaluated the effectiveness of energy restricted diet among moderate alcohol drinkers (consuming one/two drinks/day) and found a reduction in body weight among overweight and obese individuals. Breslow and Smothers [14] assessed the relationship between the continuous measure of BMI and four categories of quantity of drinking and also found a linear dose-response relationship between BMI and increasing quantity of drinking alcohol. Our results were similar but provided additional information that the beneficial effect of drinking disappears beyond consuming two drinks a day and may actually result in weight gain with heavy drinking.

The frequency of drinking was inversely related to obesity in this sample. Respondents consuming alcohol more frequently were less likely to be obese. In a recent study of primary care patients attending community clinics, we [13] found a significantly lower odds of obesity among those who consumed alcohol three or more days per month as compared to non-drinkers (Adjusted OR = 0.49, *p *= 0.037). Tolstrup et al [23] studied both the quantity and frequency of drinking in a Dannish population. The odds of obesity were lower among frequent drinkers, consuming alcohol seven days a week, as compared to those drinking less frequently. Similarly, Breslow and Smothers [14] also found the lowest BMI among persons who drank small quantities regularly. The effect was primarily observed among females. In our study the association was similar among males and females. The consistency of the inverse relationship observed between obesity and frequency of drinking suggest that the beneficial effect of drinking on obesity is present when the alcohol is consumed in moderate amounts on a regular basis.

We also explored association between overweight/obesity and average volume of drinking per week. Those drinking less than five drinks per week had lower odds of obesity. It has been argued that the association between BMI and alcohol consumption is obscured when average volume is used as a measure of alcohol consumption [14]. However, our results were consistent with the results obtained using other measures of alcohol consumption in the study.

## Conclusion

The beneficial effect of moderate use of alcohol beverages on diabetes and other chronic diseases has been well established [24-27]. However, prospective epidemiological studies are needed to confirm if the same beneficial effects can be extended to the obesity. Actively promoting moderate use of alcohol as a strategy to combat obesity would be inappropriate at this early stage of our understanding about the underlying mechanisms that link alcohol use with weight control. Furthermore, it should be noted that the data give no evidence to advise non-drinkers to start drinking alcohol just for reducing body weight. However, the evidence reported here argues against a strategy of promoting complete abstention at-least among those who regularly consume alcohol.

## Competing interests

The author(s) declare that they have no competing interests.

## Authors' contributions

AAA carried out the study, performed statistical analyses and drafted the manuscript. JER participated in the design of the study and drafting of the manuscript. All authors read and approved the final manuscript.

## Pre-publication history

The pre-publication history for this paper can be accessed here:


